# Cooking of Artemide Black Rice: Impact on Proximate Composition and Phenolic Compounds

**DOI:** 10.3390/foods10040824

**Published:** 2021-04-10

**Authors:** Antonio Colasanto, Fabiano Travaglia, Matteo Bordiga, Stefania Monteduro, Marco Arlorio, Jean Daniel Coïsson, Monica Locatelli

**Affiliations:** Dipartimento di Scienze del Farmaco, Università degli Studi del Piemonte Orientale “A. Avogadro”, 28100 Novara, Italy; antonio.colasanto@uniupo.it (A.C.); fabiano.travaglia@uniupo.it (F.T.); matteo.bordiga@uniupo.it (M.B.); stefania.monteduro@uniupo.it (S.M.); marco.arlorio@uniupo.it (M.A.); monica.locatelli@uniupo.it (M.L.)

**Keywords:** anthocyanins, antioxidant activity, fiber, flavonoids, HPLC, phenolic acids, proteins, rice

## Abstract

The consumption of black rice has grown in recent years due to its particular organoleptic properties and high content of antioxidant polyphenols, which make it a sort of natural functional food. However, heat treatment applied during cooking can influence the content and the composition of antioxidant components, particularly anthocyanins, the main compounds of black rice, responsible for its color. The aim of this work was to evaluate the impact of different cooking techniques (boiling, microwaves oven, under pressure pot and risotto preparation) on the chemical and nutritional composition of the Italian Artemide black rice. Different cooking methods had significant and different impact on rice composition. Proximate composition was not affected by cooking, except for moisture, which increased, and fiber content, which decreased. Total polyphenols, total anthocyanin content, and antioxidant capacity were reduced; moreover, anthocyanins and phenolic acids determined by HPLC-DAD generally decreased, with the only exception of protocatechuic acid. The risotto preparation was the most useful cooking technique to preserve anthocyanins and antioxidant activity. Our results demonstrated the importance to study cooking methods and to evaluate their impact on rice characteristics, in order to preserve its nutritional and beneficial properties.

## 1. Introduction

Rice is one of the most produced and consumed cereals in the world. Rice crops belong to two different varieties: *Oryza glaberrima L*., which is widespread in West Africa, and *Oryza sativa L.,* the most common variety in Asia and Europe. This last variety has two major subspecies: *Oryza sativa L. spp japonica,* rife in South-East Asia, Japan, Europe, and the U.S., and *Oryza sativa L. spp indica*, which is primarily consumed in India and Southern China. Italy is the main producer of rice (*Oryza sativa L.)* in Europe; approximately 90% of its production is concentrated in two Northern regions—Piedmont and Lombardy [1]. Even though white rice varieties are the most consumed, pigmented rice became more popular in the last few years due to their antioxidant properties and phenolic content, and the potential beneficial effects on the human health [2]. The pigments that give the characteristic color (red, purple or black) to rice are mainly contained in the bran fraction. The color is visible when the grains are dehulled, but it can be removed by polishing, revealing the white endosperm [3]. Pigmented rice varieties contain more nutrients than white rice, thus providing nutritional advantages [4]. For these particular characteristics, pigmented rice, can be considered a “functional food” that, thanks to the presence of particular substances, can potentially induce health benefits in addition to the nutritional ones.

The origin of black rice is placed in the Asian continent, where it was widely used in traditional medicine [5]; it has been introduced in Italy only in the last 25 years because of the climate incompatibility, due to its photosensitivity and instability. The first variety of black rice grown in Italy was Venere rice, obtained from a hybridization process between a black rice from South-East Asia and a local variety. Although in Italy the production of pigmented rice is still limited, it is now increasing, due to the growing consumers’ appreciation [6]. At today, 23 pigmented varieties (black or red) are registered in the Italian Varietal Register (crop year 2019/2020). Among them, Artemide rice, which has been obtained through a natural hybridization process between Venere rice and a white *indica* rice variety (long and narrow grain), is characterized by high phenolic content, in particular anthocyanins, and antioxidant activity [1].

The characteristic compounds of black rice, which determined its greatest spread, are anthocyanins. They are mainly located in the pericarp of black rice and are known to be influenced by various factors, including pH, temperature, light, oxygen, and metal ions [7]. Most of the studies reported in literature observed that cyanidin-3-glucoside is the main anthocyanin in black rice, followed by peonidin-3-glucoside; in some cases, they represent more than 90% of the total anthocyanins determined [8,9].

Most of the literature concerning antioxidant phenolic compounds in rice is referred to raw samples and concerns the analysis of antioxidant capacity, as well as the quantification of individual compounds [2,10,11,12]. In contrast, the effect of cooking on the antioxidant capacity and the quali-quantitative composition of phenolic compounds has been less studied. Considering that cereals must be consumed after cooking, it is important to analyze how heat treatments can influence the antioxidant components of these foods. Several studies conducted on different food matrices have shown that thermal treatment reduces the content of phenolic compounds and the antioxidant activity. In the case of rice, it was observed a different decrease based on variety and cooking methods. Finocchiaro et al. [13] reported a higher reduction of phenolic compounds in red rice than in white varieties. Several authors evidenced that cooking process is detrimental for the polyphenolic fraction, in particular anthocyanins [14,15]. Recently, Catena et al. [16] observed a loss of total anthocyanins in the range 70%–92% for Violet Nori rice, depending on different cooking method.

The aim of this work was to evaluate the impact of cooking on the chemical and nutritional composition of the Italian Artemide rice, evaluating, in particular, the proximate composition, and the antioxidant polyphenolic compounds. Four different domestic cooking techniques were applied: boiling, microwaves, under pressure and “risotto”.

## 2. Materials and Methods

### 2.1. Chemicals

Methanol, acetonitrile (all HPLC grade), and formic acid (50%, LC–MS grade) were purchased from Sigma–Aldrich (Milan, Italy). Ultrapure water (18.2 MΩ cm at 25 °C) was obtained by ELGA PURELAB Ultra system (M-medical, Cornaredo, Milan, Italy). Anthocyanins (Cyanidin-3-O-glucoside and Peonidin-3-O-glucoside) were obtained from LGC Standard srl (Sesto San Giovanni, Milan, Italy). All the other polyphenol reference compounds (gallic acid, protocatechuic acid, *p*-hydroxybenzoic acid, vanillic acid, coumaric acid, ferulic acid, catechin, myricetin), chemicals, and reagents were of analytical grade and purchased from Merck KGaA (Darmstadt, Germany).

### 2.2. Rice Samples and Cooking Procedures

The “Artemide” black rice was supplied by the local company “Azienda Agricola Luigi e Carlo Guidobono Cavalchini, tenuta La Mondina”, located in Casalbeltrame, Novara (Italy), in under-vacuum packages kept at room temperature.

Four different domestic cooking techniques were applied: boiling (BOI), microwave oven cooking (MW), cooking in a pressure cooker (PRES), and “risotto” (RIS). Microwave oven cooking was applied in two methods, different for the soaking pre-treatment of rice, the total cooking time and the total volume of water employed. The risotto mode was instead carried out in three different ways, with or without a preliminary toasting phase and, in the first case, with or without the addition of extra-virgin olive oil. Excepting for the microwave methods, all the cooking experiments were performed using an electric ceramic hob (Electrolux PQX320C, Stockholm, Sweden); the hob was always set to the power level “3”, equivalent to a slow/medium cooking. The different cooking conditions are detailed below and resumed in Table 1.

*Boiling (BOI)*. 200 g of black rice and 1 L of distilled water were placed in a steel pot and covered with a lid. The time required for proper cooking was calculated from the moment when cooking water started boiling and was determined as 25 min. During the cooking, the rice was occasionally mixed with a wooden spoon. At the end of cooking, the rice was drained, put in a container, and left to cool at room temperature (about 20 °C), covered by a film, away from light.

*Microwave oven (MW)*. The *MW* cooking was done in two different ways:

*MW-a*: 50 g of black rice and 100 mL of distilled water were placed in a container suitable for the microwave cooking. The rice was left to soak for 1 h and 30 min, then it was cooked covered by a lid for 10 min at 600 W in a microwave oven (Dauer, model DM2). Afterward, 50 mL of water was added and the rice was cooked for further 5 min.

*MW-b*: 50 g of black rice and 150 mL of distilled water were placed in a container suitable for the microwave cooking. The rice was left to soak for 45 min, then it was cooked covered by a lid for 15 min at 600 W. Afterward, 50 mL of water was added and the rice was cooked for further 5 min.

For both the MW cooking methods, the water amount was determined in order to avoid the draining at the end of the cooking (water was completely absorbed by the rice or evaporated). The rice was put in a container and left to cool at room temperature (about 20 °C), covered by a film, away from light.

*Pressure cooker (PRES)*. 500 g of black rice and 1000 mL of distilled water were placed in a pressure cooker operating at 112 °C and 55 kPa (Lagostina Itala Control, Italy). The total cooking time was 30 min. At the end of cooking all the water was absorbed by the rice or evaporated. The rice was put in a container and left to cool at room temperature (about 20 °C), covered by a film, away from light.

*Risotto (RIS)*. The risotto cooking was done in three different ways:

*RIS-a*: 200 g of black rice and 250 mL of distilled water were placed in a cooking pan, then cooking was started, occasionally stirring with a wooden spoon. After 15 min, the water was almost completely absorbed/evaporated, then other 250 mL of water were added. The cooking was continued for further 20 min.

*RIS-b*: 200 g of black rice were placed in a cooking pan and toasted for 5 min, continuously mixing with a wooden spoon. Then, 250 mL of distilled water was added and cooking was continued, stirring sometimes. After 15 min, the water was almost completely absorbed/evaporated, so others 250 mL of water were added; the rice was left to cook for further 15 min.

*RIS-c*: 200 g of black rice and three tablespoons of commercial extra-virgin olive oil were placed in a cooking pan and toasted for 5 min, continuously mixing with a wooden tablespoon. Then, 250 mL of distilled water was added and cooking was continued, occasionally stirring. After 15 min, the water was almost completely absorbed/evaporated, so others 250 mL of water were added to finish cooking in further 15 min.

For all the RIS methods, the water was completely absorbed by the rice or evaporated, and the rice was put in a container and left to cool at room temperature (about 20 °C), covered by a film, away from light.

### 2.3. Proximate Composition

The moisture content of raw and cooked rice was determined using a Sartorius MA30 thermo-balance (Sartorius AG, Goettingen, Germany). Due to the differences of moisture in raw grains and cooked samples, all the results were expressed on a dry weight (d.w.) basis. Prior to the other analyses, the cooked samples have been freeze-dried (Heto Drywinner 8, Copenhagen, Denmark) according to the following procedure: pre-freezing −25 °C for 1 h; primary drying −10 °C for 16 h and 0 °C for 16 h; secondary drying 10 °C for 30 h and 20 °C for 10 h. Finally, both raw grains and lyophilized cooked samples were ground to a fine flour using a laboratory blender (Sterilmixer 12, International PBI, Milan, Italy). The determination of proximate composition was performed as previously described in Giordano et al. [17]. The total nitrogen content and total protein content (conversion factor: 5.95) were obtained according to the Kjeldahl method, using Kjeltec system I (Foss Tecator AB, Höganäs, Sweden). The ash content was determined after combustion of the organic material in a muffle furnace, according to the AOAC (1990) procedure. The total dietary fiber was determined by means of the Megazyme total dietary fiber analysis kit.

### 2.4. Extraction Procedure of Phenolics

100 mg of sample (raw grains or freeze-dried cooked rice, previously ground) were extracted with 1.7 mL of distilled water under agitation for 10 min at room temperature. The extraction was repeated for other two times with water and for other three times with ethanol. Each extraction step was followed by centrifugation at 9200× *g* for 5 min, then the clear supernatants were combined. The extracts were divided in aliquots for the analyses and stored at −20 °C until use. For each rice sample the extraction was performed in triplicate.

### 2.5. Total Phenolic Content

The total phenolic content (TPC) was determined according to a modified version of the Folin–Ciocâlteu method [18]. Briefly, 100 μL of Folin–Ciocâlteu reagent and 350 µL of aqueous Na_2_CO_3_ (5% *w*/*v*) were added to an appropriate volume of rice hydroalcoholic extract and then the solution was diluted to a final volume of 2900 μL with distilled water. After 1 h, the absorbance was read at 760 nm, using an Evolution 60S spectrophotometer (Thermo Fisher Scientific, Waltham, MA, USA). Results were expressed as catechin equivalents (CE) through a calibration curve.

### 2.6. Total Anthocyanin Content

The content of TA (total anthocyanins) and TMA (total monomeric anthocyanins) was determined by the pH differential method, based on the protocol described by Lavelli, Harsha, and Spigno [19]. Different samples were opportunely diluted with potassium chloride buffer (0.025 M), pH 1.0, until the absorbance of the sample at 520 nm was within the linear range of the spectrophotometer. The same dilution factor (DF) was applied to the dilution with sodium acetate buffer (0.4 M), pH 4.5. Solutions at pH 1.0 were let to equilibrate for 5 min and those at pH 4.5 for 15 min; then the absorbance was measured at both 520 and 700 nm. The concentration of TA and TMA in the extracts was expressed as cyanidin-3-O-glucoside (Cn-3-Glu) equivalents according to the equations:

TMA (μg/mL) = [(A_520 nm_ − A_700 nm_)_pH 1_ − (A_520 nm_ − A_700 nm_)_pH 4.5_] × *MW* × d × 1000/ε (1)

TA (μg/mL) = [(A_520 nm_ − A_700 nm_)_pH 1.0_] × *MW* × d × 1000/ε (2)

where *MW* is the molecular weight of cyanidin-3-glucoside (449.2 g/mol), d is the dilution factor, ε is the molar extinction coefficient of cyanidin-3-O-glucoside (26,900 M^−1^ cm^−1^). Final results were then expressed based on rice weight (d.w.).

### 2.7. Antioxidant Activity (DPPH Radical Scavenging Assay)

The DPPH radical scavenging assay was performed according to the method validated in Locatelli et al. [20]. Briefly, 700 μL of sample (rice extracts properly diluted with MeOH) or MeOH (control) were added to the same volume of methanolic solution of DPPH^•^ (100 μM). The solutions were shaken and left in the dark at room temperature for 20 min, then the absorbance was read at 515 nm. The antioxidant activity of rice extracts was expressed as inhibition percentage of the radical. The antioxidant activity was finally expressed as Trolox equivalents (TE) by means of a calibration curve.

### 2.8. RP-HPLC-DAD Analysis

A Shimadzu LC-20A Prominence chromatographic system equipped with a diode array detector (DAD detector SPD-M20A) was used. Separation was performed on a reversed-phase Synergi TM 4 μm Max-RP 80 Å LC Column (250 × 4.6 mm i.d., with particle size of 4 μm) (Phenomenex, Torrance, CA, USA), protected by a guard column containing the same phase, at 30 °C. The mobile phase consisted of water/formic acid/acetonitrile (87:10:3, v/v) (eluent A) and water/formic acid/acetonitrile (40:10:50, v/v) (eluent B) using the following program gradient: from 6 to 20% B (20 min), from 20 to 40% B (15 min), from 40 to 60% B (5 min), from 60 to 90% B (5 min), isocratic 90% B (5 min), from 90 to 6% B (0.5 min), isocratic 6% B (22.5 min). Total run time was 73 min, at a constant flow rate of 500 μL/min. The injection volume was 5 μL. Cyanidin-3-O-glucoside and peonidin-3-O-glicoside were tentatively identified by comparison with retention times of individual authentic standard molecules and their UV–Vis spectra; the quantification was performed on the basis of calibration curves obtained with the corresponding standards. Cyanidin-3-O-rutinoside and peonidin-3-O-rutinoside were identified based on chromatographic characteristics determined in our previous study [1] and were quantified as glucoside equivalents. In the Supplementary material file, the chromatogram of the standards and, as an example, the chromatogram of a “risotto” rice sample are reported. For each reference compound, calibration curves at six different concentration levels were obtained. Details concerning the method validation (concentration range, *R*^2^, LOD, LOQ) are reported in Bordiga et al. [21]. Polyphenolic rice extracts were centrifuged (14,000 rpm for 20 min, microcentrifuge 5417R, Eppendorf, Milan, Italy) prior to the injection in the chromatographic system.

### 2.9. Statistical Analysis

Results were expressed as mean ± standard deviation (SD) of at least three independent experiments. Differences were estimated by analysis of variance (ANOVA) followed by Tukey’s honest significant difference test. The statistical significance level was set to 0.05. All statistical analyses were performed using the free statistical software R 4.0.0 version [22].

## 3. Results and Discussion

The impact of cooking on the chemical and nutritional composition of the Italian Artemide black rice was evaluated by testing different domestic cooking techniques, in particular, boiling, microwaves (2 different conditions), under pressure and “risotto” (three different conditions). Cooking parameters were selected considering different points. First, the amounts of rice were chosen to simulate realistic homemade cooking, with quantities (from 50 g to 500 g) comparable to 1 or more portions. The ratios rice/water were optimized to allow a complete absorption of the water by the rice, except for boiling. Cooking times were selected in order to guarantee a similar texture of the rice, independently from the cooking method. For microwave cooking two different soaking times were used, in order to evaluate the impact of this pre-treatment. For risotto cooking toasting phase (5 min) was also considered, with and without the use of extra virgin olive oil.

All samples were placed into a container and left to cool at room temperature (about 20 °C), covered by an aluminum foil, to ensure the stabilization of the rice weight, and it was possible to proceed with the determination of moisture and lyophilization, after which the other analyses were carried out.

### 3.1. Proximate and Phenolic Composition of Uncooked Artemide Black Rice

In the first part of the work, the proximate and phenolic composition of uncooked black Artemide rice was determined. The moisture content was measured as 11.7%, total dietary fiber, proteins, and ashes were 10.8%, 10.5%, and 1.95% on dry weight (d.w.), respectively (Table 2). While black rice moisture content is comparable with that of both polished and whole grain white rice (about 12%), total dietary fiber and protein contents are higher in Artemide black rice. Indeed, considering that rice pigments are mainly located in the external layers of the grain (bran), in order to preserve the color, Artemide rice have to be consumed (and analyzed) in the whole grain form, thus maintaining high nutritional value. In particular, Artemide rice showed a fiber content higher than polished white rice (about 1.15% d.w.), than whole white (or brown) rice (2.16% d.w.), and also than Venere black rice (5.8 % d.w.) [23]. In the same manner, the protein content of Artemide rice is higher compared to polished white rice (7.6% d.w.), brown rice (8.5% d.w.), and Venere rice (8.9% d.w.) [23].

Whole grain cereals are also a good source of antioxidant compounds, mostly polyphenols [24]. Pigmented rice, from this point of view, is particularly interesting because its color is correlated to the presence of anthocyanins, and their content is highly correlated with the antioxidant capacity [25,26]. The anthocyanins’ content in rice was analyzed in many studies, especially focused on black pigmented rice; in some red varieties, in fact, the pigmentation is not due to the presence of anthocyanins. Despite the wide number of black rice varieties characterized in the literature, their composition resulted quite similar. In fact, all the studies reports that cyanidin-3-O-glucoside is the main anthocyanin, followed by peonidin-3-glucoside [1,8,9,25,27,28,29]. This is confirmed for the Artemide black rice analyzed in this work; cyanidin-3-O-glucoside was the highest content anthocyanin (3623 ± 126 µg/g d.w.), accounting for about 89% of the identified most abundant anthocyanins, followed by peonidin-3-glucoside (323 ± 9 µg/g d.w.). Total phenolic content, total antioxidant activity, total and monomeric anthocyanins and phenolic compounds quantified in this work are higher than those obtained in our previous studies [30]. Although the rice was obtained by the same producer and was cultivated in the same geographic area (Novara province, Northern Italy), changes related to cultivation year, climatic conditions, parasites or weed attach, and/or different agronomic practices, could have had an impact on the phenolic compounds’ biosynthesis and their final concentration in rice.

### 3.2. Proximate Composition of Cooked Artemide Rice

In the second part of the work, the proximate composition of cooked Artemide rice was evaluated, in order to check the influence of different cooking methods on its nutritional characteristics. The results are summarized in Table 3 and, except for moisture, expressed on a dry weight basis (d.w.). Moisture content increased in all the cooked samples, with an average rise of 3.84 times the value of the content in raw rice (11.7%), due to the water absorption during the cooking process. The values ranged from 52.7% (RIS-a) to 65.4% (PRES); the moisture content was lower than 60%, excepting for PRES, probably due to the higher temperature reached, resulting in more complete pasting of starch granules. The “risotto” modes showed the lowest values, while boiling and MW produced similar values; not significantly (*p* > 0.05) differences were observed based on the different soaking times prior to the *MW* cooking. In fact, the moisture absorption during soaking is generally described by a curve tending to saturation, in which the maximum moisture levels correspond to the maximum water sorption capacity [31].

Concerning proteins and ashes content, no significant (*p* > 0.05) differences were found between cooking methods (average values of 10.4% and 1.89%, respectively) and the values were statistically similar to those observed in raw rice (10.5% and 1.95%, respectively), therefore suggesting that cooking methods do not involve variations in the number of proteins and ashes.

Regarding total dietary fiber, in all the cooked samples a significant (*p* < 0.001) decrease compared to the uncooked rice (10.8%) was observed, probably because cooking led to the degradation of some fiber components. The higher fiber content has been observed in RIS-c sample (9.23%), while the lower in RIS-b sample (7.16%).

### 3.3. Phenolic Composition of Cooked Rice

#### 3.3.1. Total Phenolic Content, Total Anthocyanins and Antioxidant Activity of Cooked Artemide Rice

**Figure 1:**
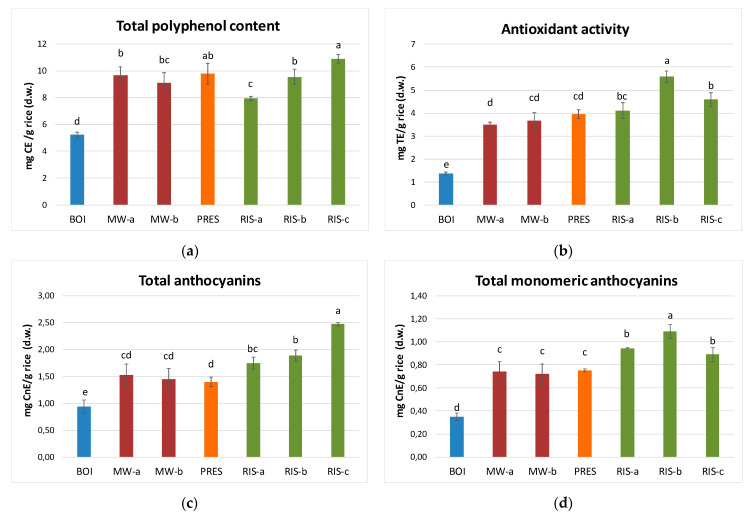
(**a**)Total phenolic content (mg CE/g d.w.), (**b**) total antioxidant activity (mg TE/g d.w.), (**c**) total anthocyanins (mg CnE/g d.w.), and (**d**) total monomeric anthocyanins (mg CnE/g d.w.) quantified in cooked black rice, expressed as mean ± standard deviation. For each parameter, values with different letters are significantly different (*p* < 0.05). CE: catechin equivalents; CnE: cyanidin-3-O-glucoside equivalents; TE: Trolox equivalents.

The total phenolic content (TPC) of cooked rice samples was expressed as milligrams of catechin equivalents (CE) per gram of rice (dry weight, d.w.) (Figure 1, panel A). The results showed that the TPC of cooked samples is significantly (*p* < 0.001) lower than in raw black rice. The thermal treatment applied during cooking determined the degradation of most phenolic compounds, which on average decrease by about 83%. The greatest loss occurred in the boiling method (about 90% decrease). This type of cooking is the only one in which there is no complete absorption of the water; therefore, it can be assumed that polyphenolic compounds solubilized in the cooking water were lost in the discarded exceeding water, as previously observed by Melini et al. [6]. The other cooking methods showed more similar results among each other. Comparing the different RIS samples, RIS-a showed the greater loss of total phenolic content (−85% respect to the uncooked rice). This suggest that the toasting phase, carried out in the other RIS methods and typically performed in the traditional “risotto” preparation to reduce the starch release during cooking, could produce a sort of barrier on the grain, able to reduce also the loss of phenolic compounds. Moreover, RIS-c sample showed the higher TPC (corresponding to a reduction of 79% respect to raw rice), and this could be related to the use of extra virgin olive oil, a polyphenols rich matrix, during toasting.

The total anthocyanins content of cooked black rice was determined as both total anthocyanins (TA) and total monomeric anthocyanins (TMA) (Figure 1, panels C and D, respectively), and the values were expressed as cyanidin-3-O-glucoside equivalents (mg CnE/g d.w.). As for TPC, also for TA and TMA it was observed a significant (*p* < 0.001) decrease in cooked samples compared to raw rice (on average −77% for TA and −81% for TMA). Moreover, in this case, the greatest loss occurred in BOI sample: the TA values decreased from 6.99 µg CnE/g in raw black rice to 0.94 µg CnE/g in BOI, and from 4.23 µg CnE/g in raw black rice to 0.35 µg CnE/g in BOI for TMA content, thus recording a reduction of 87% and 92%, respectively. No statistical differences were found between MW-a, MW-b and PRES cooking, with an average decrease of TA and TMA of 79% and 83%, while RIS cooking provided the highest anthocyanins’ content, with a decrement of TA and TMA on average of 71% and 77%, respectively. In a general way, TMA were subjected to a stronger degradation in respect to TA, which include all types of anthocyanins/anthocyanidins and polymeric forms; these results confirm our previous observations on Artemide rice, i.e., that monomeric anthocyanins are less stable to thermal treatment than non-monomeric forms [31] and are also in agreement with previous literature data [32]. Furthermore, while RIS-c sample showed the higher TA content (2.47 mg/g d.w., loss of 65%), RIS-b was the most TMA-rich sample (1.09 mg/g d.w., loss of 74%). We suggest that this difference could be related to a higher heating temperature in RIS-c due to the oil presence during toasting, thus resulting in a higher degradation of TMA in RIS-c than in RIS-b.

The antioxidant activity was evaluated by the DPPH^•^ radical scavenging assay, a common and easy method, useful to made comparison among samples, even if not exhaustive for the determination of the total antioxidant capacity. The results, expressed as Trolox equivalents (TE) (Figure 1, panel B), showed a trend quite similar to that observed for TMA content. RIS samples showed greater results (4.11, 5.59, and 4.60 mg TE/g d.w. for RIS-a, RIS-b, and RIS-c, respectively), with an average loss of 78% compared to raw black rice (21.4 mg TE/g d.w.); as expected, the most significant (*p* < 0.001) loss occurred in BOI (94% decrease). The high correlation evidenced between the antioxidant activity and TMA content (*p* < 0.001, r = 0.973), suggest a significant contribution of this class of phenolic compounds to the antiradical properties of rice.

#### 3.3.2. Individual Anthocyanins

The content of individual monomeric anthocyanins in cooked Artemide rice was determined through RP-HPLC-DAD analysis; results are reported in Table 4 and expressed as μg/g (d.w.). Three main anthocyanins were identified through the comparison with the elution times of the standard molecules (in the supplementary materials, by way of example, the chromatograms of a rice sample are reported); the most abundant was cyanidin-3-O-glucoside, accounting for slightly less than 90% in all the samples, followed by peonidin-3-O-glucoside and cyanidin-3-O-rutinoside. Peonidin-3-O-rutinoside, determined only in minor quantities in raw rice (10.2 μg/g d.w.), was not quantified after cooking. BOI rice showed the lowest concentrations and was also characterized by the highest loss compared to raw rice (−97% for Cn-3-glc and Pn-3-glc and −96% for Cn-3-rut). These data agree with the spectrophotometric results, even if the relative reduction determined for the individual compounds is higher than that observed for TMA. Differently to the spectrophotometric determinations, MW rice cooked in different conditions showed some significantly differences each other. In MW-a, the longer soaking time (1 h and 30 min against 45 min for MW-b) combined with a shorter cooking time (15 min against 20 min for MW-b) allowed to maintain higher values of both Cn-3-glc and Pn-3-glc content. PRES cooking produced results similar to MW-b cooking regarding the first two anthocyanins but evidenced a higher reduction of Cn-3-rut concentration (with a loss of 94% compared to raw black rice). In a general way, RIS cooking proved to be the best method for anthocyanins preservation, with a general average loss of 89%. Interesting results emerged comparing the different RIS methods; in particular, anthocyanins have been better preserved in RIS-b, with an average loss of 86%. As previously mentioned, we suggest that the toasting phase, a poorly investigated topic, in RIS-b can contribute to create a “barrier” reducing the loss of anthocyanins, while the adding of extra virgin olive oil in RIS-c could determine a major degradation of anthocyanins, due to a higher thermal impact.

Bhawamai et al. [33] showed for black rice a loss of about 55% of anthocyanins and about 67% of Cn-3-glc after cooking in a rice cooker for 25 min, measuring 1.0–1.2 mg anthocyanins and 238–296 µg Cn-3-glc content per gram in dry cooked rice. These authors observed a loss in anthocyanins lower than that observed in the present work; this could be due to the different cooking method, but also to the specific characteristics of black rice employed, in fact Artemide rice presented a higher anthocyanin content, both before and after cooking.

#### 3.3.3. Phenolic Acids and Flavonoids

Beside anthocyanins, other phenolic compounds were identified and quantified in cooked rice; the results, expressed as μg/g (d.w.), are summarized in Table 5. In a general way, a decrease of concentrations compared to uncooked rice was observed also for individual phenolic compounds. The only one exception is protocatechuic acid, which showed a significant (*p* < 0.001) increase in almost all the cooked samples, resulting as the most abundant among the identified compounds. In fact, this phenolic acid can derive by the scission of flavylium cation of cyanidin-3-glucoside during cooking [29]. The same effect was observed after thermal treatment of microencapsulated anthocyanin-rich extracts, used as functional ingredient in baked model biscuits [30]. The higher increase was observed in PRES sample (+60% respect to uncooked rice), while the lower in MW samples, in particular in MW-b, for which the increase respect to the concentration in raw rice (+8%) was not statistically significant (*p* > 0.05). No differences were found between RIS-a, RIS-b and RIS-c samples, evidencing an average increase of 20%. Differently to the cooking methods involving the water absorption, the protocatechuic acid concentration in BOI rice was significantly (*p* < 0.01) lower (−61%) than in uncooked rice. It is important to note that the low values observed in BOI sample should not be related to a reduced degradation of cyanidin-3-glucoside; more probably, the protocatechuic acid formed from the anthocyanin scission was dissolved in the cooking water and was eliminated with it. In fact, all the identified phenolic compounds were determined in the lowest concentrations in BOI sample.

Following protocatechuic acid, gallic acid, vanillic acid, and catechin are the three phenolic compounds present in higher amount in all the cooked samples; RIS-b sample showed the highest concentrations of them: 27.0, 28.7, and 26.6 μg/g d.w., respectively.

Interestingly, myricetin was the compound less affected by the thermal degradation. Excluding boiling (in which a loss due to water discarding necessarily occurred), reduction during cooking ranged from −39% in MW-a to −14% in RIS-b; in PRES sample the myricetin content did not statistically varied in respect to the cooked rice. In respect to these results, it would be interesting to know if part of myricetin could derive from the degradation of other compounds and/or the release from glycosylated forms during cooking (and the application of pressure in PRES cooking could justify this hypothesis), or if it is simply more stable than the other compounds. Myricetin was already identified in black rice as aglycone [34], but also as the corresponding 7-O-glucoside derivative [35].

## 4. Conclusions

Four different types of cooking (boiling, microwaves, under pressure and risotto preparation) were tested in this work to evaluate their impact on the chemical and nutritional composition of Italian Artemide black rice. Cooking procedures were optimized and standardized in the laboratory, in order to simulate realistic homemade cooking. The risotto mode was the best cooking method to preserve antioxidant capacity, polyphenols, and anthocyanin content, while boiling turned out to be the worst, due to the fact that part of polyphenolic compounds remained in the exceeding cooking water.

The main novelty in our work is to improve the knowledge about pigmented rice, such as Artemide rice, which requires a longer cooking procedure than white rice. The data obtained were not limited to polyphenols variations [36], but we have also improved the information about the cooking impact on the main nutrients.

In conclusion, different cooking methods had significant and different impact on rice composition, suggesting the importance of evaluating the treatment of rice before consumption, in order to limit the loss of antioxidant phenolic compounds. Based on these results, it would be interesting to evaluate also other cooking methods and treatments of rice, at both domestic and industrial level. Other future research could be focused on rice digestive process, in particular exploiting in vitro simulated methods, in order to evaluate the fate of polyphenolic and anthocyanic compounds along the oro-gastrointestinal tract, and to estimate their bioaccessibility and bioavailability.

## Figures and Tables

**Table 1:** Cooking conditions applied in the experiments.

Cooking Procedure	Ratio Rice/Water (g/mL)	Cooking Time (min)	Notes
Boiling (BOI)	200/1000	25	Time calculated from the boiling of water
Microwave oven (MW-a)	50/150	15	Previous soaking time: 1 h and 30 min
Microwave oven (MW-b)	50/200	20	Previous soaking time: 45 min
Pressure cooker (PRES)	500/1000	30	-
Risotto (*RIS-a*)	200/500	35	-
Risotto (*RIS-b*)	200/500	35	Time including 5 min toasting
Risotto (*RIS-c*)	200/500	40	Time including 5 min toasting in extra-virgin olive oil

**Table 2:** Proximate composition, total phenolic content (mg CE/g d.w.), total antioxidant activity (mg TE/g d.w.), anthocyanins (mg Cn-3-Glu/g d.w.) and individual phenolic compounds (µg/g d.w.) determined in uncooked Artemide rice. The results are expressed as mean ± standard deviation.

Composition of Uncooked Artemide Rice	
Moisture (%)	11.7 ± 0.4
Proteins (% d.w.)	10.5 ± 0.2
Total dietary fibre (% d.w.)	10.8 ± 1.4
Ashes (% d.w.)	1.95 ± 0.09
Total phenolic content (mg CE/g d.w.)	51.8 ± 2.2
Total anthocyanins (mg CnE/g d.w.)	6.99 ± 0.53
Total monomeric anthocyanins (mg CnE/g d.w.)	4.23 ± 0.45
Antioxidant activity (mg TE/g d.w.)	21.4 ± 0.1
*Anthocyanins* (µg/g d.w.)	
Cyanidin-3-O-glucoside	3623 ± 126
Cyanidin-3-O-rutinoside	107 ± 5
Peonidin-3-O-glucoside	323 ± 9
Peonidin-3-O-rutinoside	10.2 ± 0.2
*Other phenolic compounds* (µg/g d.w.)	
Gallic acid	86.5 ± 0.9
Protocatechuic acid	85.8 ± 0.8
*p*-hydroxybenzoic acid	4.80 ± 0.18
Vanillic acid	91.8 ± 0.2
Coumaric acid	7.29 ± 0.29
Ferulic acid	9.36 ± 0.76
Catechin	47.9 ± 2.6
Myricetin	8.52 ± 0.49

CE: catechin equivalents; CnE: cyanidin-3-O-glucoside equivalents; TE: Trolox equivalents.

**Table 3:** Proximate composition of cooked Artemide rice; the results are expressed as mean ± standard deviation. Values with different letters in the same column are significantly different (*p* < 0.05).

	Moisture (%)	Proteins (% d.w.)	Total Dietary Fiber (% d.w.)	Ashes (% d.w.)
BOI	57.0 ± 0.2b	10.2 ± 0.2a	7.91 ± 0.27bc	1.99 ± 0.10a
MW-a	56.8 ± 0.4b	10.4 ± 0.1a	8.54 ± 0.81bc	1.85 ± 0.06a
MW-b	56.2 ± 0.8b	10.2 ± 0.2a	8.28 ± 0.05bc	1.83 ± 0.09a
PRES	65.4 ± 0.2a	10.4 ± 0.1a	7.55 ± 0.50bc	1.99 ± 0.06a
RIS-a	52.7 ± 0.4d	10.6 ± 0.3a	7.24 ± 0.11bc	1.86 ± 0.06a
RIS-b	53.9 ± 0.4c	10.5 ± 0.7a	7.16 ± 0.80c	1.87 ± 0.07a
RIS-c	53.7 ± 0.03cd	10.3 ± 0.3a	9.23 ± 0.59ab	1.84 ± 0.03a

**Table 4:** Anthocyanins (µg/g d.w.) identified in cooked black rice; the results are expressed as mean ± standard deviation. Values with different letters in the same column are significantly different (*p* < 0.05).

	*Cn-3-glc* *(µg/g d.w.)*	*Pn-3-glc* *(µg/g d.w.)*	*Cn-3-rut* *(µg/g d.w.)*
BOI	118 ± 1 ^e^	10.5 ± 0.3 ^f^	4.73 ± 0.48 ^c^
MW-a	293 ± 40 ^c^	26.9 ± 1.4 ^bc^	11.1 ± 0.1 ^b^
MW-b	238 ± 7 ^d^	21.2 ± 1.1 ^de^	10.2 ± 1.0 ^b^
PRES	218 ± 2 ^d^	19.8 ± 0.9 ^e^	6.53 ± 0.14 ^c^
RIS-a	358 ± 7 ^b^	31.2 ± 0.3 ^b^	10.7 ± 0.8 ^b^
RIS-b	472 ± 21 ^a^	44.6 ± 2.2 ^a^	15.0 ± 0.8 ^a^
RIS-c	292 ± 7 ^c^	25.2 ± 1.2 ^cd^	9.87 ± 0.2 ^b^

Cn-3-glc, cyanidin-3-O-glucoside; Pn-3-glc, peonidin-3-O-glucoside; Cn-3-rut, cyanidin-3-O-rutinoside.

**Table 5:** Phenolic acids and flavonoids (µg/g d.w.) identified in cooked black rice; the results are expressed as mean ± standard deviation. Values with different letters in the same column are significantly different (*p* < 0.05). Values marked with # are not significantly different from the corresponding ones determined in uncooked rice. Gal, gallic acid; Proto, protocatechuic acid; Van, vanillic acid; Coum, coumaric acid; Fer, ferulic acid; Cat, catechin; Myr, myricetin.

	*Gal* *(µg/g d.w.)*	*Proto* *(µg/g d.w.)*	*Van* *(µg/g d.w.)*	*Coum* *(µg/g d.w.)*	*Fer* *(µg/g d.w.)*	*Cat* *(µg/g d.w.)*	*Myr* *(µg/g d.w.)*
BOI	6.80 ± 0.10 ^d^	33.4 ± 0.1 ^e^	6.67 ± 0.32 ^e^	1.91 ± 0.16 ^c^	-	9.22 ± 0.30 ^d^	3.32 ± 0.29 ^e^
MW-a	19.4 ± 0.8 ^bc^	96.4 ± 1.2 ^cd^	21.1 ± 0.005 ^c^	2.01 ± 0.01 ^b^	3.81 ± 0.02 ^b^	15.2 ± 2.0 ^cd^	5.20 ± 0.05 ^d^
MW-b	18.5 ± 0.2 ^c^	92.9 ± 2.0 ^d^ #	18.4 ± 0.5 ^d^	1.98 ± 0.13 ^b^	11.6 ± 0.3 ^a^	13.6 ± 0.3 ^cd^	6.28 ± 0.31 ^c^
PRES	19.6 ± 0.6 ^bc^	137 ± 1.9 ^a^	24.1 ± 0.8 ^b^	2.83 ± 0.01 ^a^	4.34 ± 0.20 ^b^	23.0 ± 3.0 ^ab^	8.72 ± 0.25 ^a^ #
RIS-a	22.1 ± 0.2 ^b^	101 ± 1.0 ^bc^	22.9 ± 0.4 ^bc^	2.12 ± 0.17 ^b^	4.34 ± 0.01 ^b^	17.6 ± 0.6 ^bc^	6.44 ± 0.36 ^bc^
RIS-b	27.0 ± 2.1 ^a^	103 ± 3.2 ^bc^	28.7 ± 0.7 ^a^	1.96 ± 0.06 ^b^	3.93 ± 0.13 ^b^	26.6 ± 1.4 ^a^	7.27 ± 0.15 ^b^
RIS-c	20.0 ± 0.03 ^bc^	104 ± 0.6 ^b^	20.7 ± 1.1 ^cd^	2.83 ± 0.19 ^a^	4.42 ± 0.23 ^b^	23.2 ± 1.1 ^ab^	7.02 ± 0.15 ^bc^

## Data Availability

Data sharing not applicable.

## Supplementary Materials

The following are available online at https://www.mdpi.com/article/10.3390/foods10040824/s1, Figure S1: Chromatogram of anthocyanins standard (cyanidine-3-O-glucoside and peonidin-3-O-glucoside) obtained by RP-HPLC-DAD (wavelength of detection: 520 nm), Figure S2: Chromatogram of anthocyanins obtained by RP-HPLC-DAD from a “risotto” rice sample (wavelength of detection: 520 nm). Cyanidin-3-O-rutinoside and peonidin-3-O-rutinoside were identified on the basis of chromatographic characteristics determined in our previous study [1]; peonidin-3-O-glucoside was not detected in cooked sample.
