# Supplementary material for: Cooking of Artemide Black Rice: Impact on Proximate Composition and Phenolic Compounds
Source: Foods. 2021 Apr 10;10(4):824. doi: 10.3390/foods10040824 (PMC8068815; doi:10.3390/foods10040824)
Supplement: Supplementary file 1 [file foods-10-00824-s001.pdf]

Supplementary Material

Cooking of Artemide black rice: impact on proximate composition and phenolic compounds

Antonio Colasanto, Fabiano Travaglia, Matteo Bordiga, Stefania Monteduro, Marco Arlorio, Jean Daniel Coïsson* and Monica Locatelli

Dipartimento di Scienze del Farmaco, Università degli Studi del Piemonte Orientale "A. Avogadro", Novara, Italy

*Correspondence: jeandaniel.coisson@uniupo.it; Tel.: +39 0321 375773; Fax: +39 0321 375621

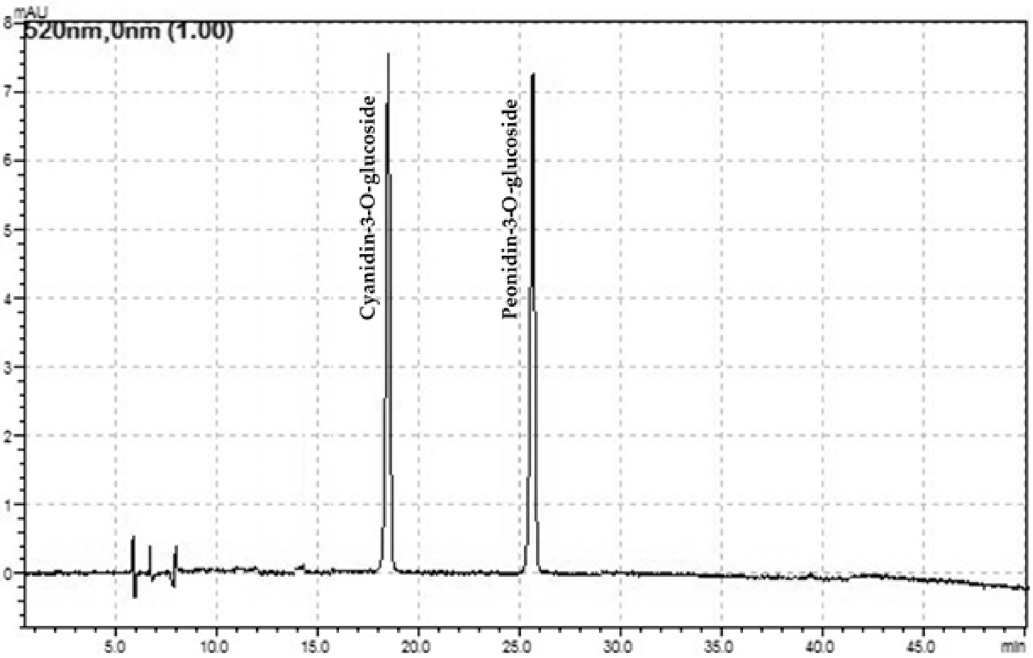

Figure 1. *Chromatogram of anthocyanins standard (cyanidine-3-O-glucoside and peonidin-3-O-glucoside) obtained by RP-HPLC-DAD (wavelength of detection: 520 nm).*

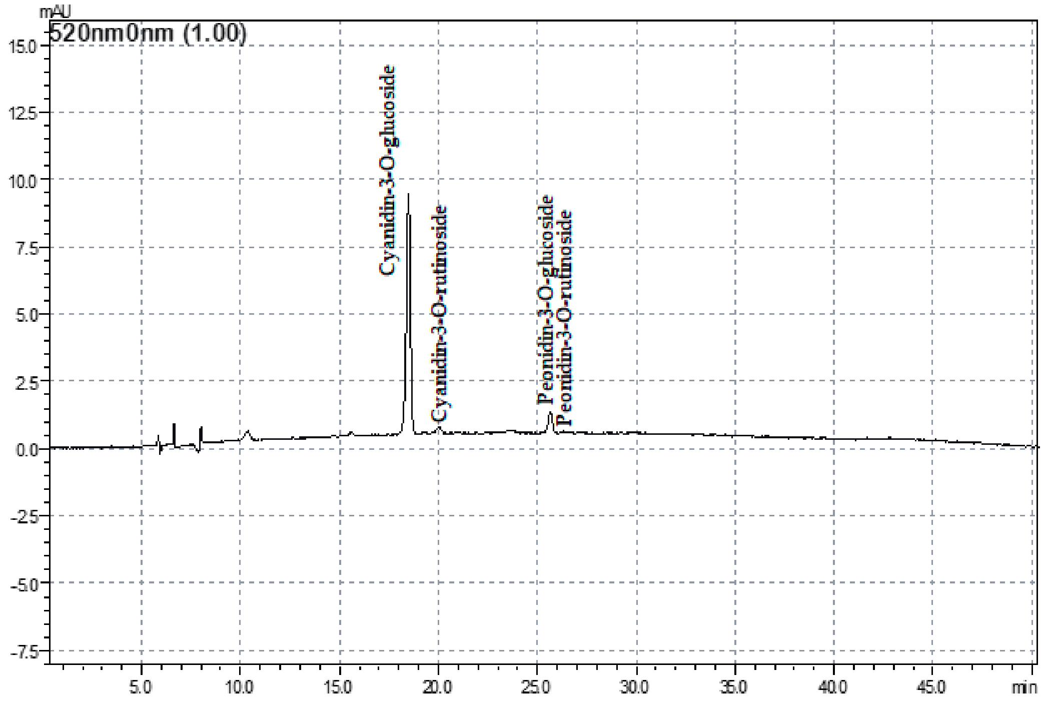

Figure 2. *Chromatogram of anthocyanins obtained by RP-HPLC-DAD from a "risotto" rice sample (wavelength of detection: 520 nm). Cyanidin-3-O-rutinoside and peonidin-3-O-rutinoside were identified on the basis of chromatographic characteristics determined in our previous study [1]; peonidin-3-O-glucoside was not detected in cooked sample.*

[1] Bordiga, M.; Gomez-Alonso, S.; Locatelli, M.; Travaglia, F.; Coïsson, J.D.; Hermosin-Gutierrez, I.; Arlorio, M. Phenolics characterization and antioxidant activity of six different pigmented Oryza sativa L. cultivars grown in Piedmont (Italy). Food Res. Int. 2014, 65, 282-290, https://doi.org/10.1016/j.foodres.2014.03.007.
